# Design of Multiplexers for IoT-Based Applications Using Stub-Loaded Coupled-Line Resonators

**DOI:** 10.3390/mi14101821

**Published:** 2023-09-23

**Authors:** Muhammad Idrees, Sohail Khalid, Muhammad Abdul Rehman, Syed Sajid Ullah, Saddam Hussain, Jawaid Iqbal

**Affiliations:** 1 Department of Electrical Engineering, Riphah International University, Islamabad 45210, Pakistan; 2 Department of Information and Communication Technology, University of Agder (UiA), N-4898 Grimstad, Norway; 3 School of Digital Science, Universiti Brunei Darussalam, Jalan Tungku Link, Gadong BE1410, Brunei; saddamicup1993@gmail.com

**Keywords:** couple line, diplexer, triplexer, open-circuit stub, bandpass filter, wireless communication

## Abstract

This paper presents the design of microstrip-based multiplexers using stub-loaded coupled-line resonators. The proposed multiplexers consist of a diplexer and a triplexer, meticulously engineered to operate at specific frequency bands relevant to IoT systems: 2.55 GHz, 3.94 GHz, and 5.75 GHz. To enhance isolation and selectivity between the two passband regions, the diplexer incorporates five transmission poles (TPs) within its design. Similarly, the triplexer filter employs seven transmission poles to attain the desired performance across all three passbands. A comprehensive comparison was conducted against previously reported designs, considering crucial parameters such as size, insertion loss, return loss, and isolation between the two frequency bands. The fabrication of the diplexer and triplexer was carried out on a compact Rogers Duroid 5880 substrate. The experimental results demonstrate an exceptional performance, with the diplexer exhibiting a low insertion loss of 0.3 dB at 2.55 GHz and 0.4 dB at 3.94 GHz. The triplexer exhibits an insertion loss of 0.3 dB at 2.55 GHz, 0.37 dB at 3.94 GHz, and 0.2 dB at 5.75 GHz. The measured performance of the fabricated diplexer and triplexer aligns well with the simulated results, validating their effectiveness in meeting the desired specifications.

## 1. Introduction

The rapid proliferation of miniaturized portable devices has ushered in a transformative era within the communications system industry, facilitating the realization of superior electrical performance benchmarks. In the context of various communication systems such as radar systems, cellular phones, and satellites, multiplexers and diplexers assume pivotal roles. The imperative to meet the exigent demands of advanced communication systems, characterized by their pervasive reach, presents a formidable challenge. Among multiplexers, diplexers represent the fundamental building block, enabling the concurrent operation of two transmitters, each functioning on distinct frequency channels, via a shared antenna. Diplexer technologies find diverse applications, prominently featuring in base stations and radio handsets tailored to cater to various cellular radio system standards. The contemporary landscape of multiservice and multiband mobile communication systems necessitates the development of diplexers and triplexers, spanning a broad spectrum of frequencies. In recent years, a myriad of diplexers, triplexers, and multiplexers have emerged, leveraging diverse topologies such as stepped impedance resonators (SIRs), coupled lines, open/shorted stub-loaded resonators (SLRs), squared open-loop resonators (SOLRs), substrate-integrated waveguides (SIWs), stub-loaded square ring resonators (SLSRRs), composite right/left-handed (CRLH) resonators, and defected ground structures (DGSs), among others. However, this study delves into the innovative design of triplexers using T-shaped short-circuited resonators [1], E-stub-loaded composite right/left-handed resonators [2], and the SSIL platform [3]. Furthermore, a lowpass-bandpass diplexer was advanced in [4] to effectively isolate the transitional frequency and local oscillator signals. Compactly designed diplexers are expounded upon in [5,6,7], while Refs. [8,9] introduce methods for crafting highly selective and isolation-optimized diplexers. On a different note, a novel approach to diplexer and triplexer design is posited in [10], eliminating the need for ancillary matching circuits by employing an open/shorted SLR. Confronting the inherent complexities of multiplexers with more than two channels, Ref. [11] proposes resonator-based microstrip diplexer and triplexer designs. Ref. [11] delineates the construction of a diplexer for 5.8 GHz ISM and WIMAX applications, alongside triplexers catering to the C-band, WIMAX, and the digital communication system (DCS). On a related tangent, Ref. [12] presents an innovative paradigm, combining frequency division, frequency selection, and power division functions within a highly integrated multifunctional diplexer, reducing the circuit footprint and bolstering system integration. Moreover, Ref. [13] introduces a novel microstrip model for designing bandpass filters using coupled stepped impedance resonators, augmenting the arsenal of design methodologies. In [14], a unique microstrip diplexer harnessing H-shaped resonators with compact dimensions and exceptional band isolation is presented, targeting applications in the industrial scientific medical (ISM) and GPS frequency bands. Addressing the perennial challenge of matching schemes, works such as [15,16,17,18] proffer systematic approaches, albeit necessitating supplemental matching circuits outside of the filters, often involving additional lumped components. Ref. [19], however, presents a highly effective microstrip diplexer utilizing compact-sized coupled SOLR-based bandpass filters, characterized by low insertion loss, high selectivity, and outstanding isolation, offering advantages in various wireless communication applications, including WiMAX. Moreover, Ref. [20] illustrates a highly selective and isolation-optimized diplexer achieved through mixed electromagnetic coupling, while Ref. [21] advocates for a dual-mode resonator-based high-isolation SIW diplexer. In tandem, Ref. [22] combines two wideband filters based on SLSRR to form a compact wideband diplexer, offering flexibility in tailoring the passband bandwidth. Catering to LTE applications, Ref. [23] unveils a novel diplexer design employing new-coupled CRLH resonators, and Ref. [24] delineates a microstrip diplexer hinging on open/short coupled lines to yield dual passbands. Akin to this, Ref. [25] presents the successful fabrication of a diplexer circuit using an FR4 substrate, yielding commendable passband characteristics. Furthermore, the annals of microstrip diplexer design exhibit an array of innovative techniques, including those based on spiral structures [26], coupled lines [27], dual-mode coupled lines [28], and magnetically coupled lines [29]. In addition, Ref. [30] employs loop resonators and lumped capacitors to feed in a triplexer design, albeit facing challenges in terms of passband insertion loss and channel isolation. Ref. [31] presents a substrate integration suspended line technology-based triplexer design, featuring favorable insertion loss and high band-to-band isolation, albeit necessitating multiple substrate layers via holes. Ref. [32] embraces tri-mode resonators and lumped elements in its triplexer design, achieving commendable in-band insertion loss and band-to-band isolation at the expense of introducing complexities such as the inclusion of a defected ground plane (DGS). Conversely, Ref. [33] explores interdigital lines on the top metal layer and complementary split-ring resonators on the ground plane for triplexer design, achieving low insertion loss while grappling with diminished isolation.

In response to the burgeoning demand for efficient multiplexers capable of managing multiple frequency bands in wireless communication applications, this paper focuses on the synthesis and design of microstrip-based multiplexers, with particular emphasis on a diplexer and triplexer operating at 2.55 GHz, 3.94 GHz, and 5.75 GHz. The diplexer design incorporates five transmission poles (TPs), strategically placed to enhance isolation and selectivity between the two passband regions. Similarly, the triplexer design utilizes seven transmission poles to ensure optimal performance across all three passbands. To evaluate the performance of the proposed designs, a comprehensive comparison was conducted with previously reported designs, considering parameters such as size, insertion loss, return loss, and isolation between frequency bands. Fabrication of the diplexer and triplexer was carried out on a compact Rogers Duroid 5880 substrate. The experimental results demonstrate low insertion losses at the specified frequencies: 0.3 dB at 2.55 GHz and 0.4 dB at 3.94 GHz for the diplexer, and 0.3 dB at 2.55 GHz, 0.37 dB at 3.94 GHz, and 0.2 dB at 5.75 GHz for the triplexer. These measured results closely align with the simulated performance, validating the effectiveness of the proposed design in meeting the desired specifications.

## 2. Methodology

### 2.1. Synthesis of the Proposed Diplexer

**Figure 1:**
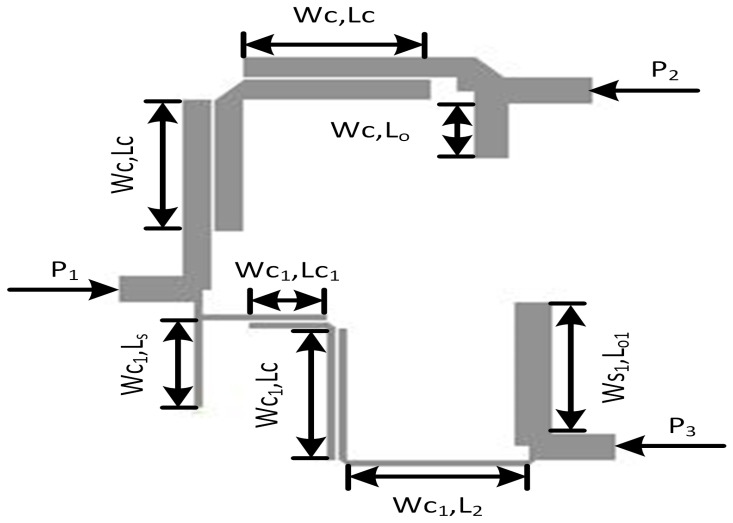
Layout model of proposed diplexer filter.

**Figure 2:**
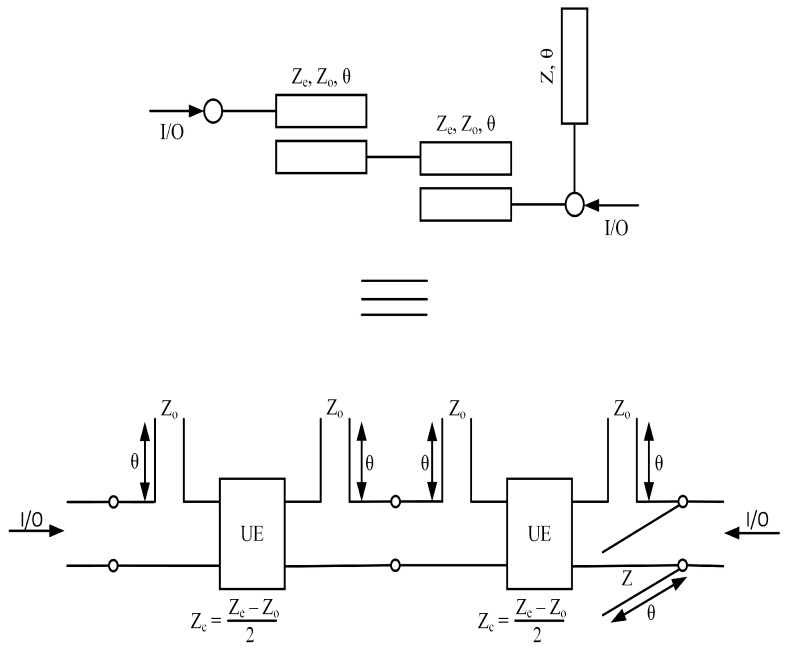
Schematic of $BPF_{1}$ with its equivalent circuit model.

This research paper introduces a new approach to the design of a microstrip diplexer, which typically consists of two bandpass filters (BPFs). The layout Model of Proposed Diplexer is Presented in Figure 1 In this study, we propose a new configuration where two BPFs are cascaded while maintaining isolation between them. The design of these bandpass resonators employs a coupled line structure. Figure 2 shows the first BPF with its distributed equivalent circuit model. It is composed of an open stub-loaded coupled lines resonator (OSLCLR). The characteristic impedance and electrical length of the coupled lines are represented as $\left(Z_{e},Z_{o},\theta \right)$, whereas (Z,θ) applies to OCS. The filtering function is extracted using the distributed equivalent circuit model. Simply multiplying all the transfer matrices of cascaded sections provide the filter’s overall transfer matrices.

Equation (1) shows the transfer matrix of the short-circuited stub’s shunt connected to a couple of lines.

(1) $$\begin{array}{c} {\left[T\right]}_{BPF1}=\prod_{q=1}^{1}\left(\begin{array}{cc} 1 & 0\\ \frac{-1}{jz\cot(\theta )} & 1 \end{array}\right)\times \prod_{q=1}^{2}\left(\begin{array}{cc} \cos(\theta ) & jz_{c}\sin\left(\theta \right)\\ \frac{j\sin(\theta )}{z_{c}} & \cos(\theta ) \end{array}\right)\times \prod_{q=1}^{4}\left(\begin{array}{cc} 1 & \frac{-jz_{o}}{\tan(\theta )}\\ 0 & 1 \end{array}\right) \end{array}$$

After determining the transfer function using Equation (1), the transmission coefficient $S_{21}$ is calculated using the known relationship illustrated in Equation (2). From transmission coefficient $S_{21}$ [34,35], the filtering function can be extracted using Equation (3).

(2) $$\begin{array}{c} S_{21}=\frac{2}{A+B+C+D} \end{array}$$

(3) $$\begin{array}{c} {\left|S_{21}\left(j\omega \right)\right|}^{2}=\frac{1}{1+\varepsilon^{2}G_{N}^{2}\left(\theta \right)} \end{array}$$

Here, $G_{N}\left(\omega \right)$ indicates the filtering function of the $N_{th}$ order, and ε is the ripple level. The derived filtering function of the proposed filter is of the order (N=6).

(4) $$G_{6}\left(\theta \right)=\frac{A\cos^{6}\left(\theta \right)-B\cos^{4}\left(\theta \right)+C\cos^{2}\left(\theta \right)-D}{\sin^{3}\left(\theta \right)}$$

(5) $$\begin{array}{c} A=\left(2\;z^{2}+z\mathrm{ze}-z\mathrm{zo}+4\;z+\mathrm{ze}-\mathrm{zo}\right)\left(-\mathrm{zo}-2+2\;z+\mathrm{ze}\right)\left(2\;z^{2}+z\mathrm{ze}-z\mathrm{zo}-4\;z-\mathrm{ze}+\mathrm{zo}\right)\\ \left(-\mathrm{zo}+2+2\;z+\mathrm{ze}\right){\left(-\mathrm{zo}+2\;z+\mathrm{ze}\right)}^{2}{\left(4\;{\left(\mathrm{ze}-\mathrm{zo}\right)}^{4}z^{2}\right)}^{1/2} \end{array}$$

(6) $$\begin{array}{c} \;B=-\left(2\;z^{2}+z\mathrm{ze}-z\mathrm{zo}+4\;z+\mathrm{ze}-\mathrm{zo}\right)\left(-\mathrm{zo}-2+2\;z+\mathrm{ze}\right)\left(2\;z^{2}+z\mathrm{ze}-z\mathrm{zo}-4\;z-\mathrm{ze}+\mathrm{zo}\right)\\ \;\left(-\mathrm{zo}+2+2\;z+\mathrm{ze}\right){\left(-\mathrm{zo}+2\;z+\mathrm{ze}\right)}^{2}{\left(\mathrm{ze}-\mathrm{zo}\right)}^{4}z^{2}{)}^{1/2} \end{array}$$

(7) $$\begin{array}{c} \;C=\left(-4\;{\mathrm{ze}}^{4}+16\;{\mathrm{ze}}^{3}\mathrm{zo}+\left(-24\;{\mathrm{zo}}^{2}+112\right){\mathrm{ze}}^{2}+\left(16\;{\mathrm{zo}}^{3}-224\mathrm{zo}\right)\mathrm{ze}-4\;{\mathrm{zo}}^{4}+112\;{\mathrm{zo}}^{2}-256\right)\\ \;z^{4}-4\;\left(\mathrm{ze}-\mathrm{zo}\right)\left({\mathrm{ze}}^{4}-4\;{\mathrm{ze}}^{3}\mathrm{zo}+\left(6\;{\mathrm{zo}}^{2}-42\right){\mathrm{ze}}^{2}+\left(-4\;{\mathrm{zo}}^{3}+84\;\mathrm{zo}\right)\mathrm{ze}+{\mathrm{zo}}^{4}-42\;{\mathrm{zo}}^{2}+96\right)\\ \;z^{3}-{\left(\mathrm{ze}-\mathrm{zo}\right)}^{2}{-\mathrm{zo})}^{2}\left({\mathrm{ze}}^{4}-4\;{\mathrm{ze}}^{3}\mathrm{zo}+\left(6\;{\mathrm{zo}}^{2}-100\right){\mathrm{ze}}^{2}+(-4\;{\mathrm{zo}}^{3}+200\;\mathrm{zo}\right)\mathrm{ze}+{\mathrm{zo}}^{4}-\\ \;100\;{\mathrm{zo}}^{2}+272)z^{2}+28\;\left({\mathrm{ze}}^{2}-2\;\mathrm{zo}\;\mathrm{ze}+{\mathrm{zo}}^{2}-3.4\right){\left(\mathrm{ze}-\mathrm{zo}\right)}^{3}z+3\;{\left(\mathrm{ze}-\mathrm{zo}\right)}^{4}({\mathrm{ze}}^{2}-2\;\mathrm{zo}\;\mathrm{ze}\\ \;+{\mathrm{zo}}^{2}-13/3{){\left(\mathrm{ze}-\mathrm{zo}\right)}^{4}z^{2})}^{1/2} \end{array}$$

(8) $$\begin{array}{c} \;D=8\;(\left(\mathrm{ze}-\mathrm{zo}+2\right)\left(\mathrm{ze}-\mathrm{zo}-2\right)z^{2}+3/4\;\left(\mathrm{ze}-\mathrm{zo}-2\right)\left(\mathrm{ze}-\mathrm{zo}+2\right)\left(\mathrm{ze}-\mathrm{zo}\right)z+1/8\\ \;\;\left({\mathrm{ze}}^{2}-2\;\mathrm{zo}\;\mathrm{ze}+{\mathrm{zo}}^{2}-6\right){\left(\mathrm{ze}-\mathrm{zo}\right)}^{2}{){\left(\mathrm{ze}-\mathrm{zo}\right)}^{2}{\left(\mathrm{ze}-\mathrm{zo}\right)}^{4}z^{2})}^{1/2} \end{array}$$

**Figure 3:**
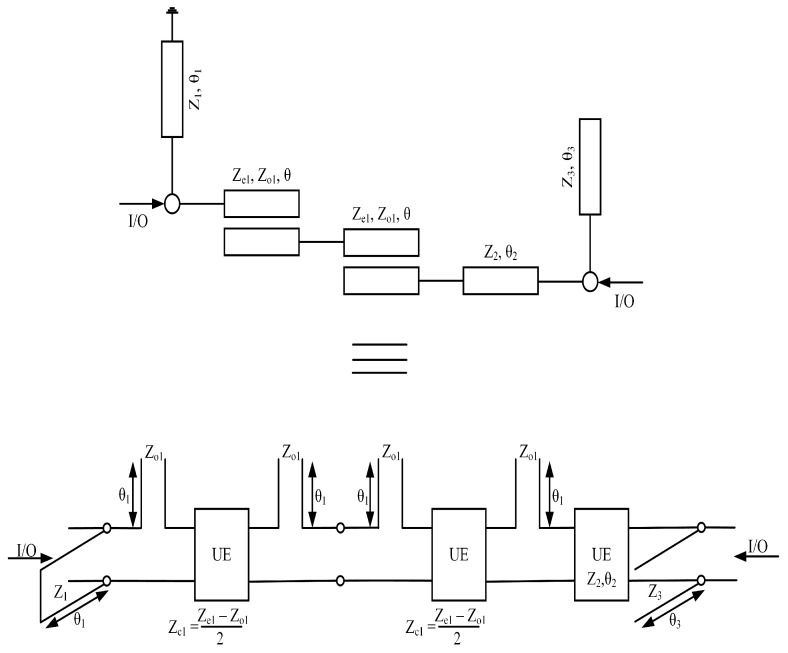
Schematic of $BPF_{2}$ with its equivalent circuit model.

In the context provided, it can be inferred that the variables *Z*, Zo, and Ze are all positive. With regards to bandpass filter $BPF_{1}$, depicted in Figure 1. $BPF_{2}$, depicted in Figure 3, is a second bandpass filter operating at 3.94 GHz; $BPF_{2}$ is composed of two coupled lines, a transmission line, short-circuit (SCS), and open-circuit stubs (OCS). Each component in the filter structure is represented by its characteristic impedance and corresponding electrical length, as depicted in Figure 2. Additionally, the equivalent circuit of the proposed filter is illustrated in Figure 2.

Equation (9) gives us transfer matrix ${\left[T\right]}_{BPF2}$ as can be seen in Figure 3:

(9) $$\begin{array}{c} \;[{\left[T\right]}_{BPF2}=\prod_{q=1}^{1}\left(\begin{array}{cc} 1 & 0\\ \frac{-1}{jz_{1}\tan\left(\theta_{1}\right)} & 1 \end{array}\right)\times \prod_{q=1}^{2}\left(\begin{array}{cc} \cos(\theta_{1}) & jz_{c1}\sin\left(\theta_{1}\right)\\ \frac{j\sin(\theta_{1})}{z_{c1}} & \cos(\theta_{1}) \end{array}\right)\times \prod_{q=1}^{1}\left(\begin{array}{cc} \cos(\theta_{2}) & jz_{2}\sin\left(\theta_{2}\right)\\ \frac{j\sin(\theta_{2})}{z_{c1}} & \cos(\theta_{2}) \end{array}\right)\\ \times \prod_{q=1}^{4}\left(\begin{array}{cc} 1 & \frac{-jz_{o1}}{\tan(\theta_{1})}\\ 0 & 1 \end{array}\right)\times \prod_{q=1}^{1}\left(\begin{array}{cc} 1 & 0\\ \frac{-1}{jz_{3}\cot\left(\theta_{3}\right)} & 1 \end{array}\right) \end{array}$$

Similarly, the procedure for the synthesis of a second bandpass filter involves evaluating the complex filtering function. Furthermore, since the two bandpass filters are parallel to each other, the overall response is obtained by multiplying the transfer functions obtained from Equations (1) and (9). This research proposes a diplexer structure consisting of two bandpass filters. In this section, the frequency response of the ideal circuit designs of $BPF_{1}$ and $BPF_{2}$ is outlined. To determine the resonance frequency of the proposed bandpass filters (shown in Figure 1 and Figure 2), appropriate values for characteristic impedances *Z* and electrical lengths θ must be selected. For $BPF_{1}$, the characteristic impedance and electrical length of the open-circuit stub (OCS) are set to Z=100.8 ohm and θ=180°, respectively. In addition, the even–odd mode structure of the coupled lines has a characteristic impedance of $Z_{o}=192.5$ and $Z_{e}=42.4$ ohm with corresponding electrical length values of θ=180°. These values (Z, Zo, Ze, and θ) produce the frequency response of an ideal $BPF_{1}$.

This reveals that the passband region ranges from 2.25 GHz to 2.86 GHz with two transmission poles within the passband. Similarly, the frequency response of $BPF_{2}$ is determined using a similar approach. For $BPF_{2}$, the characteristic impedance and electrical length of the series-coupled stub (SCS) are set to Z=Z1 and θ=45°, respectively, while the open-circuit stub (OCS) has a characteristic impedance of $Z_{1}=2Z_{3}$ and an electrical length of $\theta_{1}=2\theta =90{}^{\circ}$. The even–odd mode structure of the coupled lines has a characteristic impedance of Zo=59.2 and Ze=89.3, with corresponding electrical length values of 4θ=180°. In the pursuit of developing a diplexer, the passband filters $BPF_{1}$ and $BPF_{2}$ were interconnected in a configuration that ensures mutual isolation between the two filters. This arrangement allowed for the independent operation and functionality of each filter. The designed model of the proposed diplexer filter is illustrated in Figure 3.

**Figure 4:**
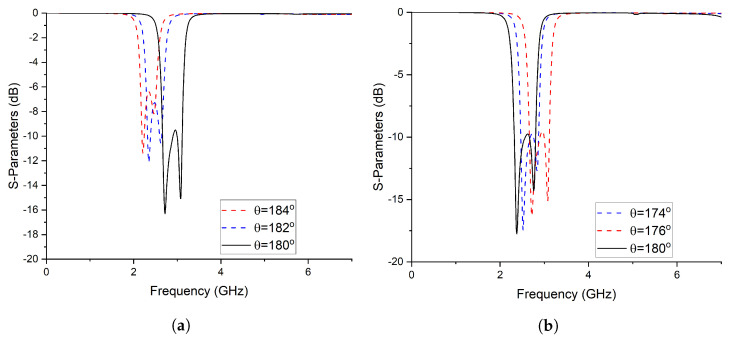
Frequency response with a variable electrical length. (**a**) By increasing the electrical length; (**b**) By decrising the electrical length.

The comprehensive analysis reveals a notable correlation between the electrical length of the coupled lines and the operating frequency [36,37]. Specifically, an increase in the electrical length of the coupled lines leads to a downward shift in the operating frequency, as demonstrated in Figure 4a. Conversely, a reduction in the physical length of the coupled lines results in a shift of the frequency spectrum towards higher frequencies, as depicted in Figure 4b.

**Figure 5:**
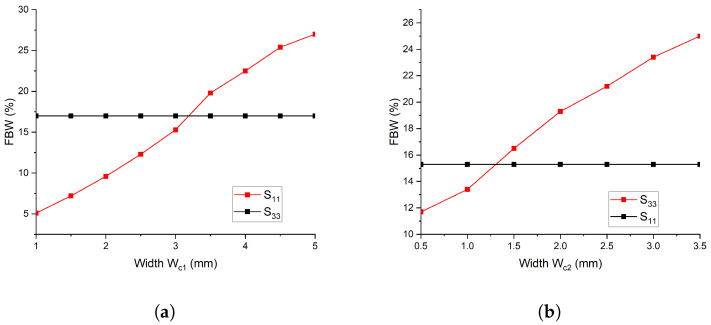
Frequency response with a variable width of coupled lines. (**a**) By Varing $W_{1}$; (**b**) By Varing $W_{2}$.

In addition to the effects of electrical length, the width of the coupled line also has an impact on the fractional bandwidth (FBW) of the first and second passbands [38,39]. This phenomenon is illustrated in Figure 5. As the width of wc1 increases, the FBW of the first passband also increases, and vice versa. However, the FBW of the second passband remains unchanged, as shown in Figure 5a. On the other hand, altering the width of wc2 of the coupled line results in a shift in the FBW of the second passband, while the FBW of the first passband remains unaltered, as depicted in Figure 5b. This phenomenon can be explained by the change in the characteristic impedance of the coupled line with variations in the width of the line, which subsequently affects the FBW of the filter. These observations are important to consider in the design of bandpass filters with desired frequency responses.

### 2.2. Synthesis of the Proposed Triplexer

**Figure 6:**
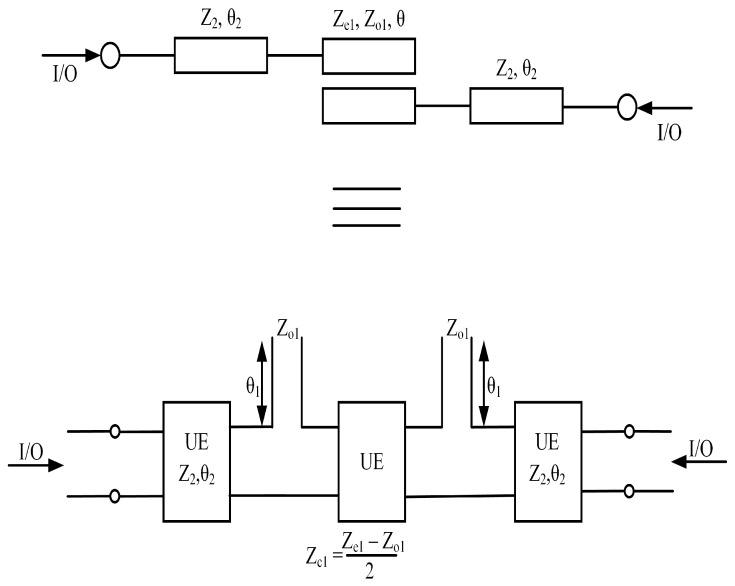
Schematic of $BPF_{3}$ with its equivalent circuit model.

This section provides the synthesis of $BPF_{3}$. Figure 6 depicts the schematic model of the proposed bandpass filter along with its equivalent circuit model. The design of this filter employs a single-stage quarter-wavelength coupled line, featuring two edge transmission lines. The even- and odd-mode characteristic impedances of the coupled lines are denoted as Ze ohm and Zo ohm, respectively, while the transmission line’s characteristic impedance is represented as $Z_{2}$. To determine the overall transfer matrices of the filter, a straightforward approach of multiplying all the cascaded sections was adopted.

(10) $$\begin{array}{c} \left[T\right]=\times \prod_{q=1}^{2}\left(\begin{array}{cc} \cos(\theta ) & jz_{1}\sin\left(\theta \right)\\ \frac{j\sin(\theta )}{z_{1}} & \cos(\theta ) \end{array}\right)\times \prod_{q=1}^{2}\left(\begin{array}{cc} \cos(\theta ) & jz_{c}\sin\left(\theta \right)\\ \frac{j\sin(\theta )}{z_{c}} & \cos(\theta ) \end{array}\right)\times \prod_{q=1}^{2}\left(\begin{array}{cc} 1 & \frac{-jz_{o}}{\tan(\theta )}\\ 0 & 1 \end{array}\right) \end{array}$$

The filtering function, denoted as $G_{4}\left(\theta \right)$, of the proposed $BPF_{3}$ is determined through the utilization of Equation (11).

(11) $$G_{4}\left(\theta \right)=\frac{\alpha \cos^{4}\left(\theta \right)+\beta \cos^{2}\left(\theta \right)+\gamma }{\sin^{3}\left(\theta \right)}$$

(12) $$\begin{array}{c} \alpha =1/4\;{\left(2\;z+\mathrm{ze}-\mathrm{zo}+z\_1\right)}^{2}\left({z\_1}^{2}-4\right){\left({\left(\mathrm{ze}-\mathrm{zo}\right)}^{2}{z\_1}^{4}\right)}^{1/2} \end{array}$$

(13) $$\begin{array}{c} \beta =-1/2\;{z\_1}^{4}+1/4\;\left(-4\;z-2\;\mathrm{ze}+2\;\mathrm{zo}\right){z\_1}^{3}-1/4\;\left(\mathrm{ze}-\mathrm{zo}+2\right)\left(\mathrm{ze}-\mathrm{zo}-2\right)\\ {z\_1}^{2}+1/4\;\left(16\;z+8\;\mathrm{ze}-8\;\mathrm{zo}\right)z\_1+2\;{\mathrm{ze}}^{2}+1/4\;\left(16\;z-16\;\mathrm{zo}\right)\mathrm{ze}+4\;z^{2}-4\;z\mathrm{zo}\\ +2\;{\mathrm{zo}}^{2}{\left({\left(\mathrm{ze}-\mathrm{zo}\right)}^{2}{z\_1}^{4}\right)}^{1/2} \end{array}$$

(14) $$\begin{array}{c} \gamma =1/4\;{z\_1}^{4}-{\left(\mathrm{ze}-\mathrm{zo}\right)}^{2}{\left({\left(\mathrm{ze}-\mathrm{zo}\right)}^{2}{z\_1}^{4}\right)}^{1/2} \end{array}$$

**Figure 7:**
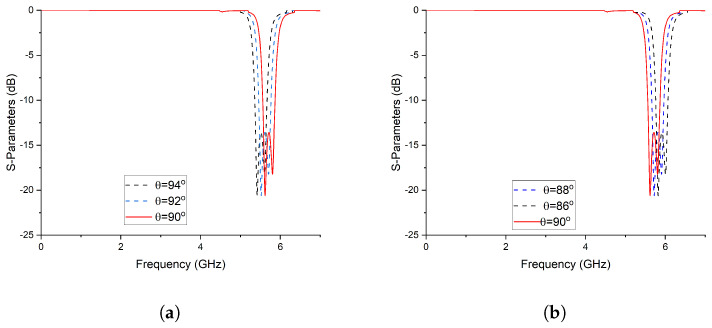
Frequency response with a variable electrical length. (**a**) By increasing the Electrical Length (**b**) By Decrising the Electrical Length.

**Figure 8:**
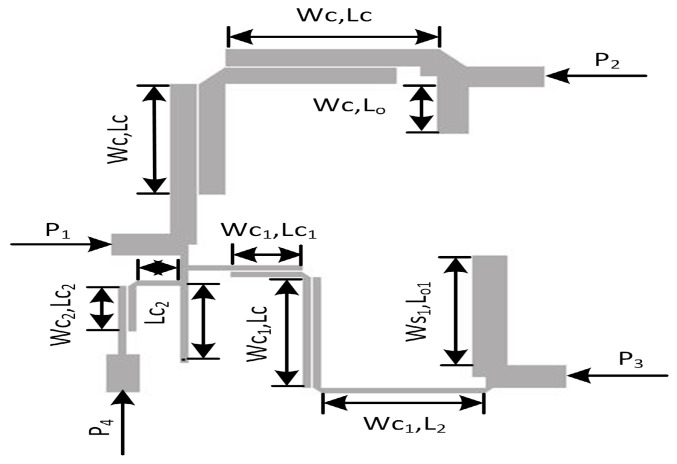
Layout model of proposed triplexer filter.

Equation (11) illustrates the filtering function associated with the topology depicted in Figure 6. In order to extract the values of the electrical parameters, the filtering function can be mapped to type 1 Chebyshev polynomials. However, the frequency-dependent term present in the denominator of the filtering function can disrupt the desired equal ripple characteristic of the Chebyshev polynomial. To mitigate this effect, a restructuring of the filtering function is proposed. To obtain the ripple factor, the filtering function is normalized. This normalized filtering function is then utilized to synthesize the filtering function, ensuring an optimal solution that addresses the aforementioned concerns. $BPF_{3}$ utilizes transmission lines with a characteristic impedance of $Z_{3}=45.09$ and an electrical length of θ=90°. The coupled lines in $BPF_{3}$ exhibit an even–odd mode structure, characterized by a characteristic impedance of $Z_{o}=67.2$ for the odd mode and $Z_{e}=157.3$ for the even mode. These modes correspond to an electrical length of 4θ=90°. In order to develop a triplexer, $BPF_{1}$, $BPF_{2}$, and $BPF_{3}$ were interconnected in a configuration that ensures mutual isolation between all three filters. This arrangement guarantees the independent operation and functionality of each filter. The detailed analysis establishes a significant relationship between the electrical length of the coupled lines and the operating frequency. It was observed that altering the electrical length of the coupled lines influences the operating frequency accordingly. Figure 7a illustrates a downward shift in the operating frequency with an increase in the electrical length of the coupled lines. Conversely, Figure 7b demonstrates a shift towards higher frequencies when the electrical length of the coupled lines is reduced. Figure 8 illustrates the designed model of the proposed triplexer filter.

## 3. Results

Due to the lossless characteristics of an ideal design, it is crucial to employ microstrip-based designs to mitigate these losses. However, microstrip circuits inherently introduce parasitic losses, which leads to variations in the performance of the microstrip diplexer when compared to the ideal circuit’s response. In microstrip design, physical parameters are derived by mapping electrical parameters from the ideal design. The initial structure of the diplexer is constructed using microstrip lines incorporating microstrip tees and bends. Bends are utilized to reduce the circuit size while preserving its electrical performance, whereas tees are employed to connect or join multiple resonator stubs and transmission lines. The dimensions of the filter, including length and width, are calculated and simulated using a line calculator. The calculated dimensions are as follows: $W_{s1}$ = 3.99 mm, Wc = 3.1 mm, $Wc_{1}$ = 0.98 mm, Lc = 19.9 mm, $Lc_{1}$ = 2.7 mm, $L_{2}$ = 8.32 mm, $L_{s}$ = 13.3 mm, $L_{o1}$ = 8.68 mm, Sc = 0.2 mm, $w_{2}$ = 1 mm, and $Lc_{2}$ = 2.7 mm.

It is evident from Figure 3 that $BPF_{1}$ is connected between port 1 and port 2; whereas $BPF_{2}$ is connected between port 1 and port 3. It can be seen that port 1 serves as an input feeding port, while port 2 and port 3 serve as output ports.

**Figure 9:**
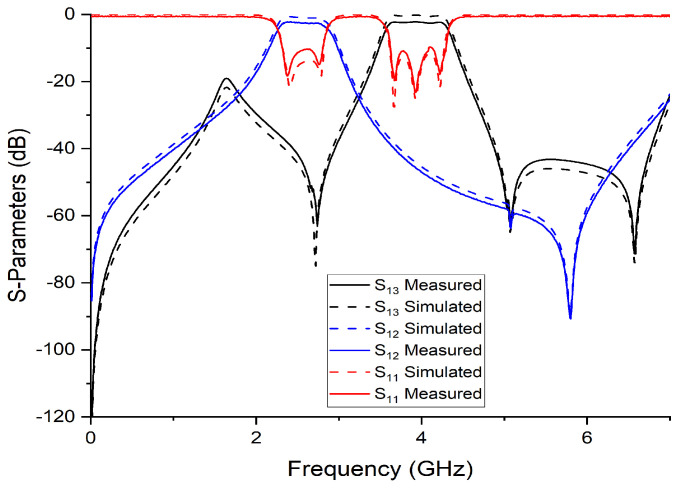
S-parameter response of proposed diplexer.

**Figure 10:**
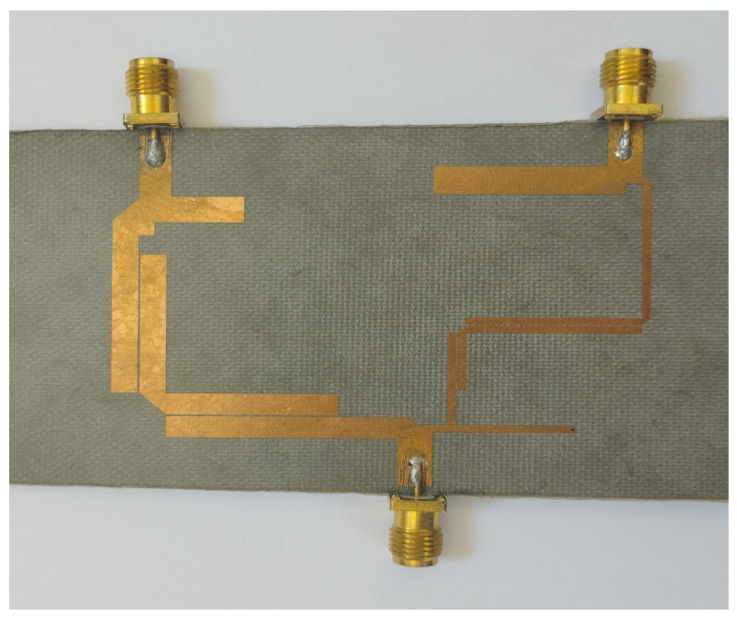
Fabricated prototype of proposed diplexer.

**Figure 11:**
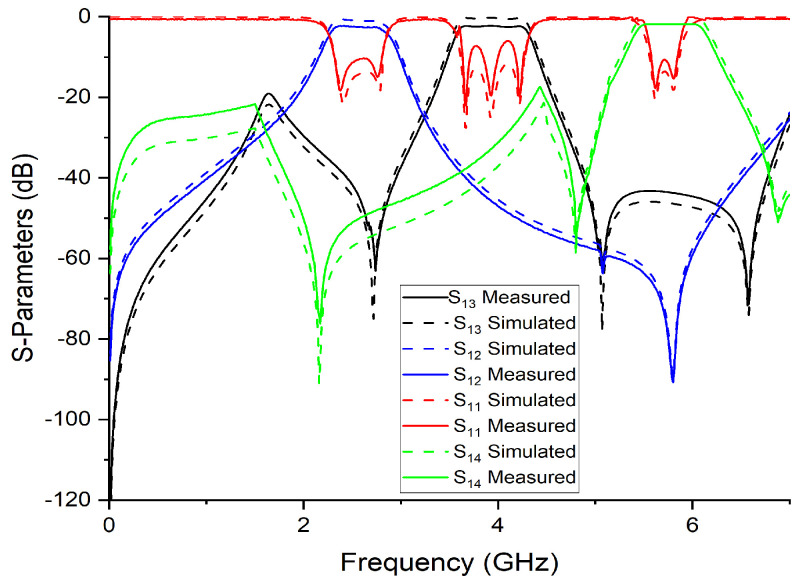
S-parameter response of proposed triplexer.

**Figure 12:**
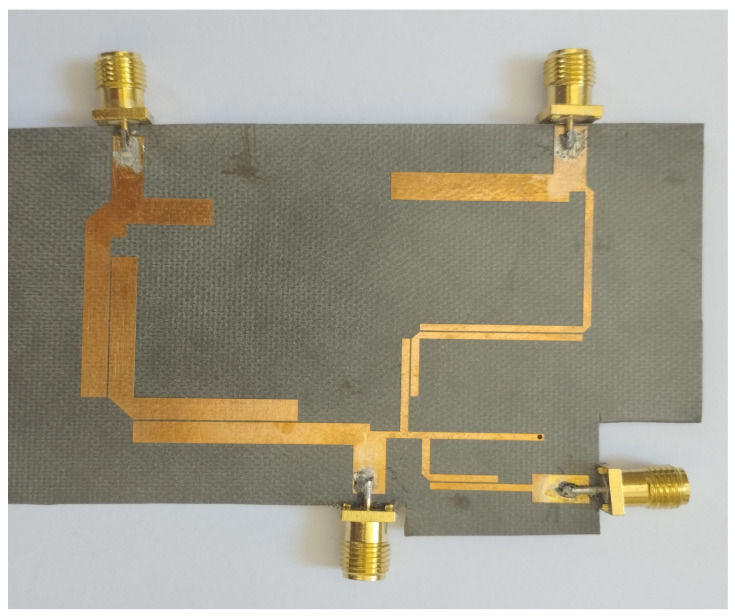
Fabricated prototype of proposed triplexer.

The other tails of $BPF_{1}$ and $BPF_{2}$ are joined together with a junction transmission line. The signal is passed on to the other two ports via port 1. Figure 9 and Figure 10 show the S-parameter response and fabricated prototype of a proposed diplexer. Two transmission poles in the first passband and three transmission poles in the second passband were achieved. They are located at 2.35 and 2.79 GHz in the first passband and 3.67, 3.93, and 4.23 GHz. In addition, by cascading the additional BSF with the diplexer, as shown in Figure 8, an additional passband was achieved at 5.75 GHz, with the fractional bandwidth of 8.6% Here, it is also noteworthy that the third BSF provides two transmission poles, which are located at 5.61 GHz and 5.87 GHz, respectively. The measured in-band return loss is greater than 11 dB. Figure 11 and Figure 12 show the S-parameter response and the fabricated prototype of a proposed triplexer, demonstrating the achievement of two transmission poles in each of the first and third passbands, and three transmission poles in the second passband. These poles are located at 2.35 GHz and 2.79 GHz in the first passband, 3.67 GHz, 3.93 GHz, and 4.23 GHz in the second passband, and 5.61 GHz and 5.87 GHz in the third passband. The triplexer exhibits three passbands centered at frequencies of 2.55 GHz, 3.94 GHz, and 5.74 GHz, with corresponding fractional bandwidths (FBW) of 23.9%, 18.2%, and 8.6%, respectively. The measured insertion loss for each band was recorded as 0.36 dB, 0.53 dB, and 0.33 dB, respectively. Moreover, the in-band return loss measured greater than 13 dB. To verify the theoretical model, the designed prototypes were fabricated on a high-frequency substrate, i.e., Rogers Duroid 5880 substrate, with ($\epsilon_{r}=2.2$, tan δ=0.0009, and height h=0.787 mm), and measured using an Agilent N5242A PNA-X for the validation of the proposed topology.

**Figure 13:**
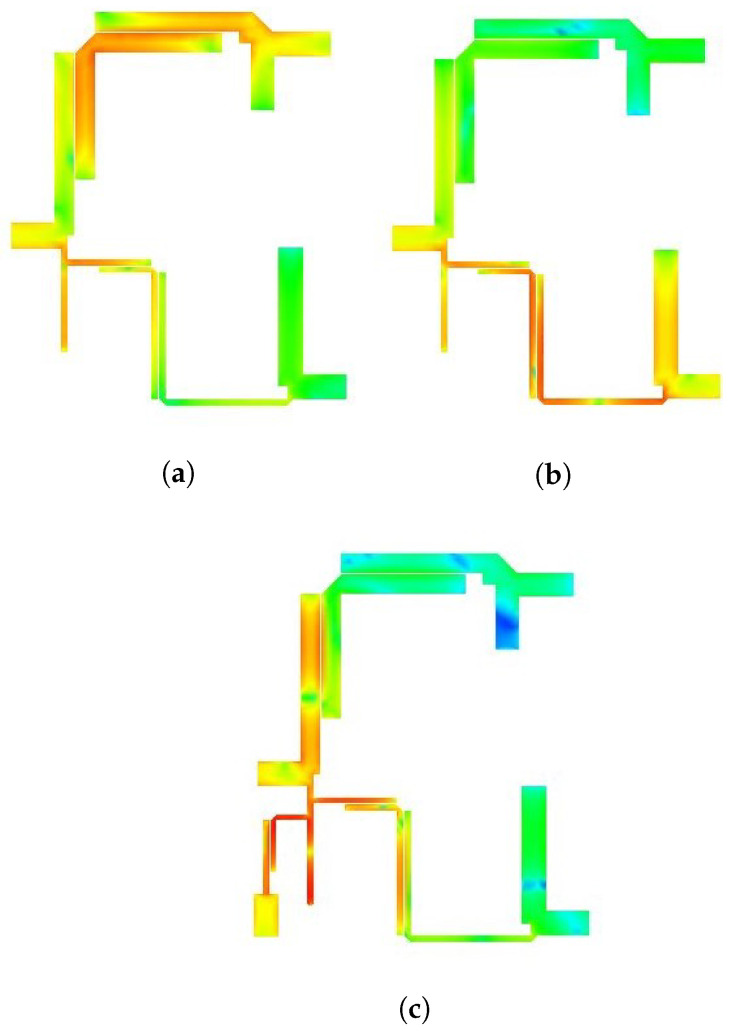
Frequency response with a variable electrical length. (**a**) $BPF_{1}$; (**b**) $BPF_{2}$; (**c**) $BPF_{3}$.

The electrical field spectrum at the central frequencies of both passbands is depicted in Figure 13. Specifically, Figure 13a displays the electric field spectrum at the frequency corresponding to the first passband, while Figure 13b,c illustrates the electric field spectrum at the frequency corresponding to the second and third passbands of the diplexer and triplexers. The simulation was conducted using a mesh size of 80 cells per wavelength and a frequency sweep of 0.01 GHz. The results clearly indicate that only $BPF_{1}$ is active at the first passband frequency, confirming the intended design concept. Conversely, at the second and third passband frequencies, $BPF_{2}$ and $BPF_{3}$ become active, further affirming the validity of the design.

The proposed design was optimized to achieve the required goals. In this section, an analysis regarding scattering parameters (S11, S12, and S13) versus the frequency is explored. Table 1 shows the empirical outcomes of all diplexer filters in accordance with the recently introduced methodology. Notably, the suggested diplexer filter demonstrates remarkable attributes of enhanced selectivity and elevated fractional bandwidth, particularly within the higher frequency ranges, while maintaining a compact circuit size.

Table 2 presents the empirical results obtained for triplexer filters using the recently introduced methodology. It is worth mentioning that the proposed triplexer filter exhibits impressive characteristics, such as improved selectivity and increased fractional bandwidth, especially in the higher frequency ranges. Remarkably, these advancements were achieved while maintaining a compact circuit size.

## 4. Conclusions

This paper presents a detailed investigation into the design and synthesis of microstrip-based multiplexers, leveraging stub-loaded coupled-line resonators to enhance their passband characteristics. Specifically, a diplexer and a triplexer were developed to operate at predetermined frequency bands. The diplexer design incorporated five transmission poles to optimize isolation and selectivity within the passband regions, while the triplexer employed seven transmission poles to ensure satisfactory performance across all three passbands. To assess the effectiveness of the proposed multiplexers, a comprehensive comparative analysis was conducted, evaluating crucial parameters such as size, insertion loss, return loss, and isolation between frequency bands. The diplexer and triplexer were fabricated on a compact Rogers Duroid 5880 substrate with ($\epsilon_{r}=2.2$, tan δ=0.0009, and height h=0.787 mm). The experimental results revealed that both multiplexers exhibited remarkably low insertion losses. Specifically, the diplexer achieved an insertion loss of 0.3 dB at 2.55 GHz and 0.4 dB at 3.94 GHz, while the triplexer attained insertion losses of 0.3 dB at 2.55 GHz, 0.37 dB at 3.94 GHz, and 0.2 dB at 5.75 GHz. Furthermore, the measured performance of the fabricated multiplexers demonstrated a strong agreement with the simulated results, affirming their efficacy in meeting the desired specifications. The proposed design, which effectively employed stub-loaded coupled line resonators, exhibits promising passband characteristics, thereby establishing its suitability for a wide range of wireless communication applications that necessitate efficient multiplexing across diverse frequency bands.

## Figures and Tables

**Table 1:** Comparison of the fabricated prototypes with the recently developed diplexers.

Ref	CF’s	TP’s	RL’s	IL	Circuit Size	FBW
	**(GH)**		**(dB)**	**(dB)**	$\left(\lambda_{g}\times \lambda_{g}\right)$	**(% Age)**
[13]	1.8/2.45	4	>19	2.16/2.24	0.31×0.61	6.27/5.44
[14]	1.2/1.6	6	>19/>25	2.4/1.7	*N/A*	7.3/11.25
[22]	1.4/2.02	6	>15	4.4/4.6	0.52×0.25	50/51.4
[25]	1.85/2.5	3	>30/>30	4.2/3.3	0.42×0.34	7.1/4.3
[26]	2.88/3.29	4	>23/>23	0.36/0.44	0.22×0.28	*N/A*
[28]	2.6/6	7	16/25	0.5/2	0.23×1.27	*N/A*
**This work**	** 2.55/3.94 **	5	**>13**	** 0.3/0.4 **	** 0.29×0.37 **	** 23.9/18.2 **

**Table 2:** Comparison of recently developed triplexers with the proposed triplexer.

Ref	CF’s	RL’s	IL	Circuit Size	FBW
	**(GH)**	**(dB)**	**(dB)**	$\left(\lambda_{g}\times \lambda_{g}\right)$	**(% Age)**
[30]	2.5/2.8/3.5	>10	3.5/2.2/2.9	0.20×0.38	4.4/2.2/3.95
[31]	0.8/2.5/5.8	>12	1.8/1.3/0.8	0.42×0.59	*N/A*
[32]	0.9/1.8/3.45	>15	1.1/1.2/1.7	0.163×0.104	22/16/9.5
[33]	1.4/1.8/3.2	>18	0.1/2/1	0.70×0.2	5.8/2.2/9.4
[34]	1.4/2.1/2.8	>14	3.6/4.3/4.8	0.19×0.11	6/6/4
**This work**	** 2.55/3.94/5.74 **	**>13**	** 0.36/0.53/0.33 **	** 0.29×0.37 **	** 23.9/18.2/8.6 **

## Data Availability

Data supporting this paper’s findings are available upon reasonable request, subject to applicable data sharing agreements and ethical considerations.
